# Cumulus cell-released tumor necrosis factor (TNF)-α promotes post-ovulatory aging of mouse oocytes

**DOI:** 10.18632/aging.101507

**Published:** 2018-07-26

**Authors:** Qiao-Qiao Kong, Jia Wang, Bin Xiao, Fei-Hu Lin, Jiang Zhu, Guang-Yi Sun, Ming-Jiu Luo, Jing-He Tan

**Affiliations:** 1 Shandong Provincial Key Laboratory of Animal Biotechnology and Disease Control and Prevention, College of Animal Science and Veterinary Medicine, Shandong Agricultural University, Tai'an City 271018, P. R. China

**Keywords:** aging, cumulus cells, FasL, oocytes, TNF-α

## Abstract

Although previous studies indicated that cumulus cells (CCs) accelerate oocyte aging by releasing soluble factors, the factors have yet to be characterized. While demonstrating that CCs promoted oocyte aging by releasing soluble Fas ligand (sFasL), our recent study suggested that CCs might secrete other factors to mediate oocyte aging as well. This study tested whether CCs accelerate oocyte aging by secreting tumor necrosis factor (TNF)-α. The results showed that mouse CCs undergoing apoptosis released soluble TNF-α (sTNF-α) during in vitro aging. While ethanol activation rates were higher, the maturation-promoting factor (MPF) activity was lower significantly after culture of cumulus-denuded oocytes (DOs) in medium conditioned with CCs for 36 h than in medium conditioned for 24 h. Aging mouse oocytes expressed TNF-receptor 1. The CCs released equal amounts of sTNF-α and sFasL during aging in vitro, and the TNF-α-knockdown CCs secreted less sFasL than the control CCs did. Treatment of DOs in vitro with sTNF-α significantly accelerated their aging. The aging-promoting effect of sTNF-α was significantly reduced in TNF-α-knocked-down CCs and in CCs from the TNF-α-knockout mice. It is concluded that mouse CCs accelerate oocyte aging by secreting sTNF-α as well as sFasL.

## Introduction

If not fertilized or activated in time after ovulation or in vitro maturation, mammalian oocytes undergo a time-dependent process of aging [1–3]. This post-ovulatory oocyte aging process has been found to affect embryo development [4–6] and offspring [7,8]. Thus, a control over post-ovulatory oocyte aging is of great importance for both normal and assisted reproduction. However, the mechanisms for post-ovulatory oocyte aging are largely unknown.

Both in vivo and in vitro matured oocytes are enclosed within cumulus cells (CCs) forming the so-called cumulus-oocyte-complexes (COCs). The CCs stay with in vivo matured oocytes for different times after ovulation in different species [9–11], but they will stay with in vitro matured oocytes until artificially removed. Previous studies have indicated that CCs accelerate oocyte aging. For example, oocytes cultured as COCs aged faster than those cultured as cumulus-denuded oocytes (DOs) [3]. Coculture with CCs or culture in medium conditioned by CCs significantly promoted aging of DOs [3,12]. The aging-promoting effect is ablated when the CCs-conditioned medium was heated to 56°C for 15 min [12]. Furthermore, apoptotic CCs accelerated porcine oocyte aging and degeneration in vitro via a paracrine manner [13]. However, although this suggests that CCs accelerate oocyte aging by releasing soluble and heat-sensitive factors, the oocyte aging-promoting factors that CCs release have yet to be characterized.

To characterize the CCs-released factors involved in promoting oocyte aging, our recent study demonstrated that CCs surrounding the aging oocytes underwent apoptosis in an time-dependent manner after ovulation and released soluble Fas ligand (sFasL), which accelerated oocyte aging by binding to Fas receptors present on the oocyte [6]. However, the medium conditioned for 24 h with H_2_O_2_-treated CCs that contained about 30 folds less sFasL showed the same or even higher capacity of inducing oocyte aging than supplementation with an optimal concentration (10 ng/ml) of sFasL [6]. Thus, the ethanol activation rate (38.3±2.7%) of DOs aging in CZB medium containing 10 ng/ml sFasL was significantly lower than that (57.0±1.2%) in DOs aging in medium conditioned with H_2_O_2_-treated CCs, which contained about 0.35 ng/ml FasL. Taken together, the results suggested that the apoptotic CCs might secrete other factors than sFasL that mediate oocytes aging as well.

The tumor necrosis factor (TNF) receptor (TNF-R) superfamily is a protein superfamily of cytokine receptors characterized by their ability to bind TNFs and induce cell apoptosis [14]. Both the TNF-R and the Fas receptor are among the important members of the TNF receptor superfamily. Studies have shown that the TNF-α system is active in ovaries of different species. For example, rat oocytes are an important source of TNF-α, and TNF-R are localized on rat oocytes, granulosa cells and interstitial cells [15]. TNF-α can have deleterious actions on bovine oocyte maturation that compromise development of the resultant embryo [16]. Mouse oocytes express both TNF-R1 and 2 and are sensitive to TNF-induced cell death [17]. Furthermore, 9-cis retinoic acid exerts its beneficial effects on pig oocyte developmental competence and embryo quality by attenuating oocyte TNF-α mRNA expression [18].

We thus hypothesized that in addition to soluble FasL (sFasL), apoptotic CCs might also secrete soluble TNF-α (sTNF-α) that act on its receptor on the oocyte to accelerate postovulatory oocyte aging. To test this hypothesis, TNF-α secretion by CCs and expression of TNF-R1 on mouse oocytes were first observed during oocyte aging; effects of treatment with sTNF-α and sFasL on the early (activation susceptibility and developmental potential) and late (fragmentation and chromosome/spindle morphology) aging manifestations of postovulatory oocytes were then compared; and finally, the role of the TNF-α signaling in promoting oocyte aging was further confirmed by knocking down TNF-α expression in CCs via RNA interference and by using TNF-α knockout (^-/-^) mice.

## RESULTS

### CCs underwent apoptosis and released sTNF-α during in vitro aging

**Figure 1:**
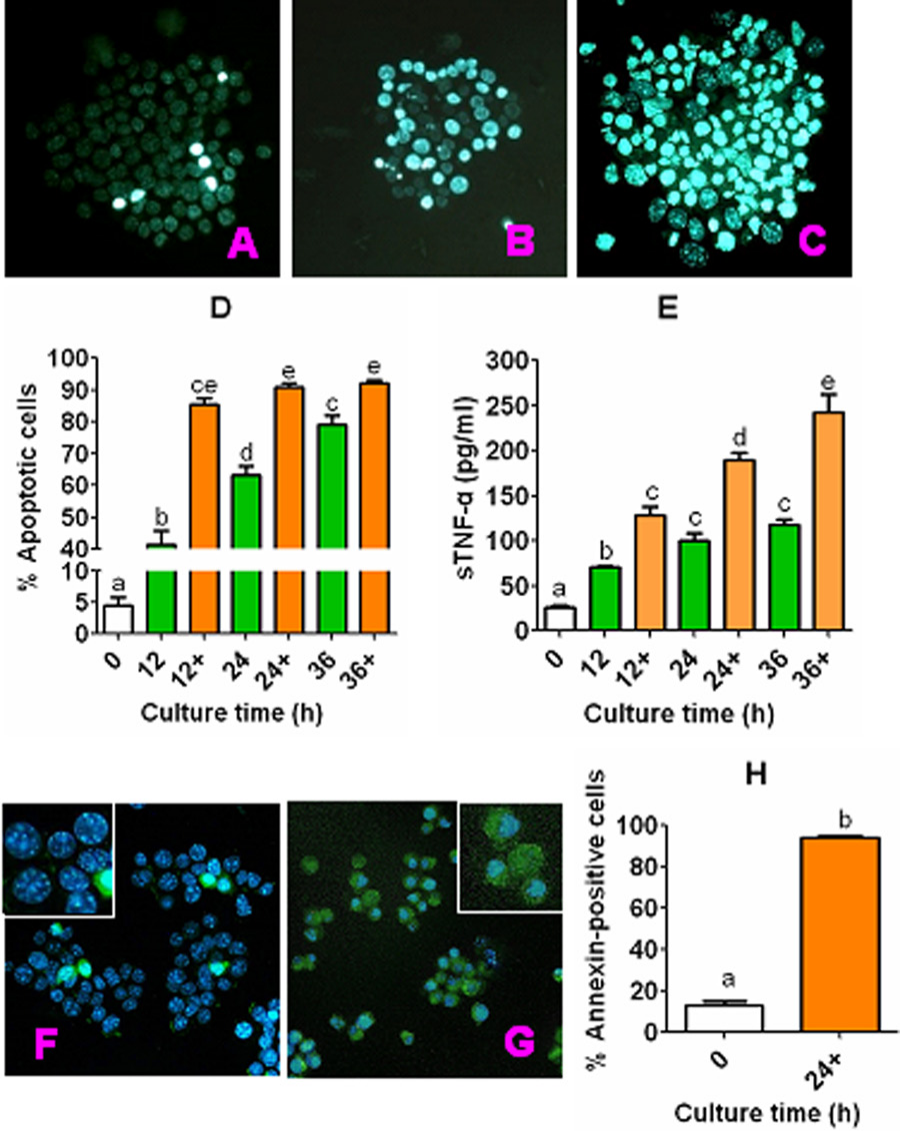
**Apoptosis and sTNF-α release during culture of CCs.** CCs recovered from newly ovulated oocytes were cultured in CZB medium alone or with 200-µM H_2_O_2_ (+). At different times of the culture, rates of apoptotic cells were observed after Hoechst or annexin-V staining, and concentrations of sTNF-α in CM were measured by ELISA. Both the Hoechst and annexin-stained smears were observed under a fluorescence microscope (Original magnification ×400). Micrographs (**A**), (**B**) and (**C**) show Hoechst-stained smears of CCs cultured for 0 h or 24 h in CZB alone or CZB with H_2_O_2_, showing approximately 6%, 54%, and 85% apoptotic cells, respectively. Micrographs (**F**) and (**G**) show Hoechst and annexin-V double-stained smears of CCs cultured for 0 and 24 h, respectively, in CZB with H_2_O_2_, showing approximately 13% and 94% apoptotic cells, respectively. The photos are merged images with Hoechst and annexin-V colored blue and green, respectively. Graphs (**D**) and (**H**) show percentages of apoptotic CCs revealed by Hoechst and annexin-V staining, respectively. Each treatment was repeated 3-4 times with each replicate containing CCs from about 60-90 freshly recovered (0 h) COCs or from one well of cultured cells. Graph E shows levels of sTNF-α in CM, and each treatment was repeated 3 times with each replicate including 100 µl of CM from one culture well. a–e: Values with a different letter above bars differ significantly (P < 0.05).

CCs recovered from newly ovulated oocytes were cultured in CZB medium alone or with 200 µM H_2_O_2_, and at different times of the culture, rates of apoptotic cells were observed following Hoechst or annexin-V staining, and concentrations of sTNF-α in the conditioned medium (CM) were measured. The results of Hoechst staining showed that percentages of apoptotic CCs increased with culture time in either CZB alone or with H_2_O_2_, and at each time point, the apoptotic percentage was significantly higher with than without H_2_O_2_ (Fig. 1A to D). The concentrations of sTNF-α also increased significantly with culture time, and were significantly higher with than without H_2_O_2_ (Fig. 1E). Our annexin-V staining (Fig. 1 F to H) further confirmed the results from Hoechst staining by showing a similar proportion between the annexin-positive and Hoechst-detected apoptotic cells in both freshly ovulated CCs (13.3 vs. 4.6%) and the CCs cultured for 24 h with H_2_O_2_ (93.9 vs. 90.8%). The results suggested that CCs underwent apoptosis and continuously released sTNF-α during in vitro aging, and that H_2_O_2_ significantly enhanced apoptosis and sTNF-α secretion in CCs.

### Expression of TNF-R1 in mouse oocytes during in vitro aging

**Figure 2:**
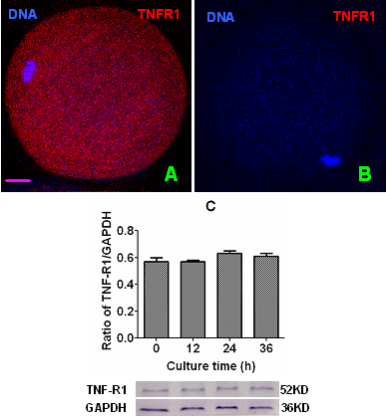
**Levels of TNF-R1 in aging oocytes.** Panels (**A**) and (**B**) are a merged image from laser confocal microscopy (original magnification ×400) showing localization of TNF-R1 in newly-ovulated oocytes. In the images, DNA and TNF-R1 were pseudo colored blue and red, respectively, and the bar is 10 µm. Image (**B**) is a negative control image showing an oocyte treated with the primary antibody against TNF-R1 omitted. Graph (**C**) shows levels of TNF-R1 (ratios of TNF-R1/GAPDH) by Western blot quantification in oocytes aging for different times in CZB medium. Each treatment was repeated 3 times with each replicate containing about 200 oocytes. Ratios of TNF-R1/GAPDH did not differ significantly (P>0.05) between culture times.

Newly ovulated DOs recovered at 13 h post hCG injection were cultured in CZB medium, and at different times of the culture, localization of TNF-R1 was observed by immunofluorescence microscopy and its quantification was conducted by Western blotting. The immunofluorescence microscopy showed that mouse oocytes contained a large quantity of TNF-R1 granules distributed on the plasma membrane and throughout the cytoplasm (Fig. 2A). The western blot quantification indicated that the intra-oocyte TNF-R1 level (ratio of TNF-R1/GAPDH) did not change significantly up to 36 h of aging in CZB medium (Fig. 2C). Together with the above results that CCs undergoing apoptosis released sTNF-α which accumulated with time during in vitro aging, our results on TNF-R1 suggested that the TNF-α system is increasingly active in aging mouse oocytes.

### Effects of culture in CM on aging of DOs

**Figure 3:**
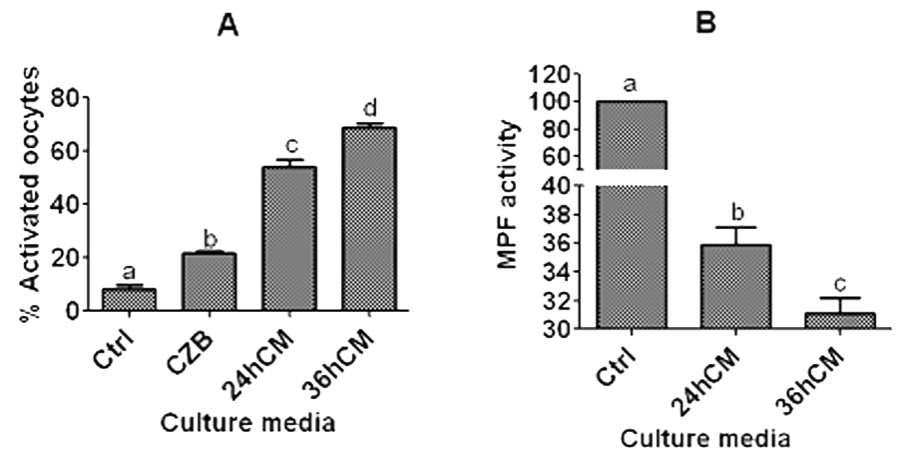
**Ethanol activation rates and MPF activity after mouse DOs collected 13 h post hCG were cultured for 12 h in different media.** To prepare CM, CCs were cultured in regular CZB medium for 24 (24hCM) or 36 h (36hCM) following a 24-h culture in CZB containing 200-µM H_2_O_2_. For controls (Ctrl), some newly ovulated oocytes were activated with ethanol or assayed for MPF activity immediately after recovery. For activation, each treatment was repeated 4-5 times and each replicate contained 25-30 DOs, and for MPF assay, each treatment was repeated 4-6 times with each replicate containing about 50 oocytes. a-d: Values with a different letter above bars differ significantly (P<0.05).

To determine that mouse CCs facilitate oocyte aging by releasing sTNF-α, mouse DOs collected 13 h post hCG were cultured for 12 h in CZB, or in CM collected at 24 or 36 h of CCs culture before ethanol activation to assess oocyte activation susceptibility or assay for MPF activity. The results showed that while activation rates were higher, the MPF activity was lower significantly after culture of DOs in 36-h CM than in 24-h CM (Fig. 3). Together with the above results that sTNF-α accumulated with increasing time of CCs culture (Fig. 1E), the results confirmed that CCs facilitated oocyte aging by releasing sTNF-α.

### Effects of culture with different concentrations of sTNF-α or sFasL on activation susceptibility and embryo developmental potential of aging DOs

**Figure 4:**
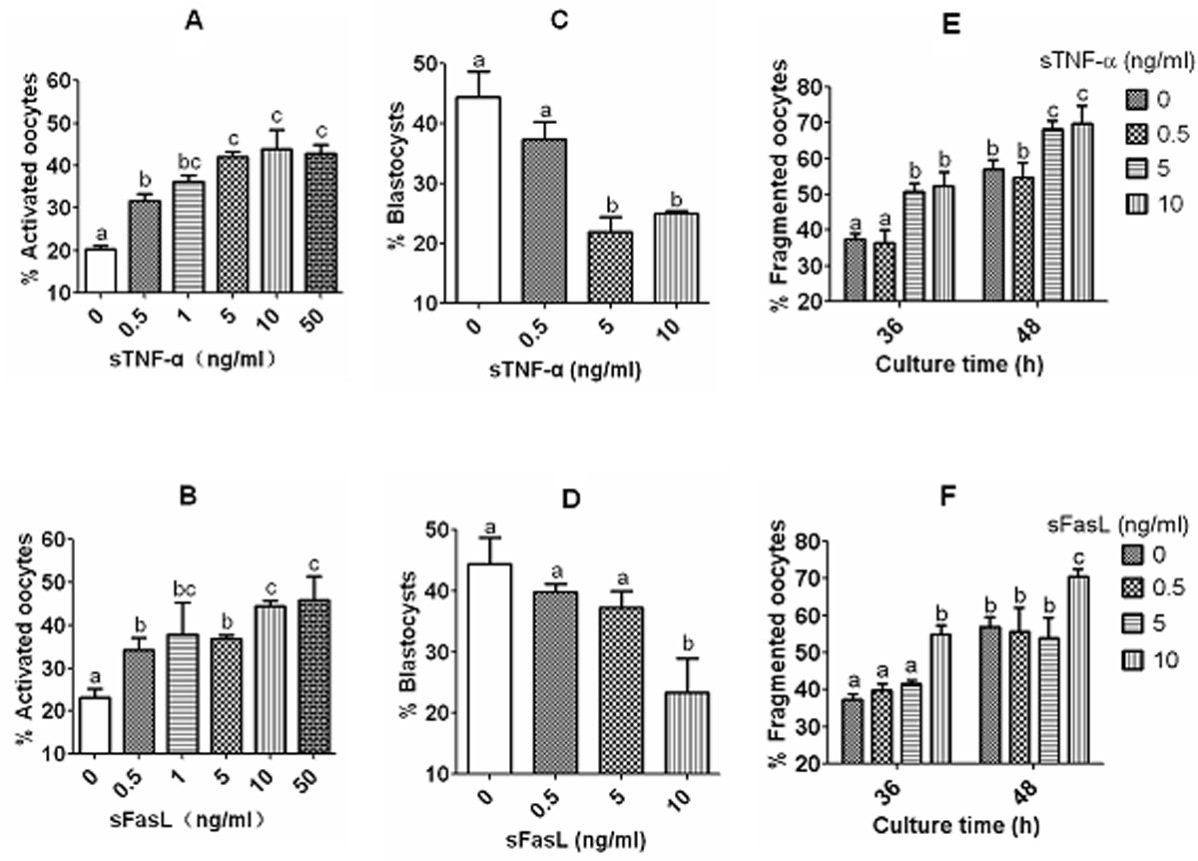
**Effects of culture with different concentrations of sTNF-α or sFasL on activation susceptibility, embryo developmental potential and cytoplasmic fragmentation of aging DOs.** While (**A**) and (**B**) show rates of ethanol-activated oocytes, (**C**) and (**D**) show blastocyst rates of Sr^2+^ activated oocytes. Mouse DOs collected at 13 h post hCG were cultured for 12 h in CZB medium containing different concentrations of sTNF-α or sFasL before activation treatment with ethanol or SrCl_2_. Each treatment was repeated 3 times with each replicate including about 30 oocytes. E and F show percentages of fragmented oocytes after newly ovulated DOs were cultured for 36 or 48 h in CZB with different concentrations of sTNF-α (**E**) or sFasL (**F**). Each treatment was repeated 4 times with each replicate containing about 30 oocytes. a–c: Values with a different letter above bars differ significantly (P < 0.05).

Mouse DOs collected 13 h post hCG were cultured for 12 h in CZB medium containing different concentrations of sTNF-α or sFasL before assessment of activation susceptibility and embryo development potential. Ethanol and Sr^2+^ activation were conducted to examine activation susceptibility and embryo development, respectively. The results showed that while activation rates increased, blastocyst rates decreased significantly with increasing concentrations of both sTNF-α and sFasL. However, while maximum ethanol activation was obtained with 5 ng/ml sTNF-α, (Fig. 4A), it was not achieved until 10 ng/ml sFasL (Fig. 4B). Similarly, while blastocyst rates decreased to the lowest level with 5 ng/ml sTNF-α (Fig. 4C), the lowest blastocyst rate was not observed until 10 ng/ml sFasL (Fig. 4D). The results suggested that both sTNF-α and sFasL promoted early manifestations of oocyte aging, and that aging mouse oocytes were more sensitive to sTNF-α than to sFasL in vitro.

### Effects of culture with different concentrations of sTNF-α or sFasL on cytoplasmic fragmentation and spindle/chromosome morphology of aging DOs

Newly ovulated DOs were cultured for 36 or 48 h with different concentrations of sTNF-α or sFasL before observation for cytoplasmic fragmentation. Oocytes with a clear moderately granulate cytoplasm, and an intact first polar body, were considered to be un-fragmented, and oocytes with more than two asymmetric cells were considered to be fragmented. When observed at either 36 h or 48 h of culture, fragmentation rates increased with both sTNF-α and sFasL concentrations. However, while percentages of fragmented oocytes increased to maximum with 5 ng/ml sTNF-α (Fig. 4E), maximum fragmentation was not observed until the concentration of sFasL increased to 10 ng/ml (Fig. 4F).

**Figure 5:**
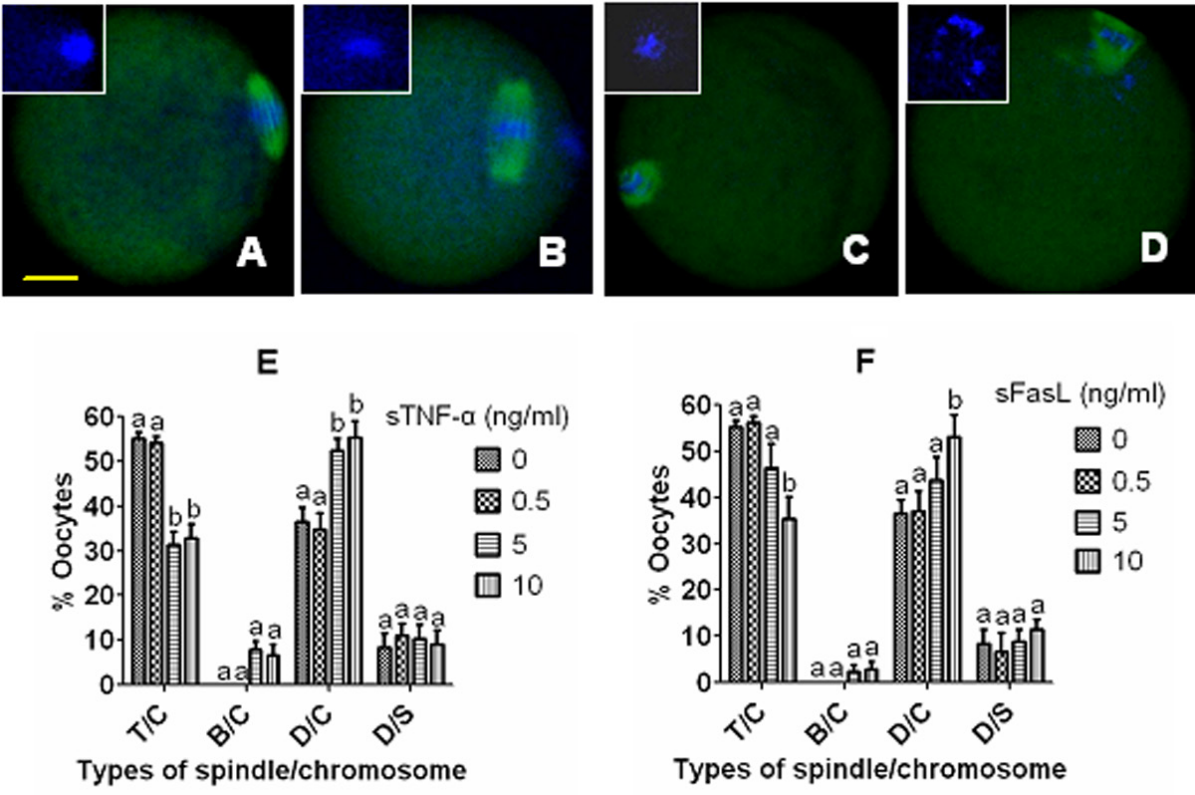
**Effects of culture with different concentrations of sTNF-α or sFasL on spindle/chromosome morphology of aging mouse oocytes.** Oocytes that had aged for 12 h in CZB containing different concentrations of sTNF-α or sFasL were cultured for 24 h in CZB medium before examination for morphology of spindles and chromosomes. In the confocal images, DNA and α-tubulin were pseudo-colored blue and green, respectively. Bar is 15 µm and applies to all images. Image (**A**) shows an oocyte with a tine-pole spindle and chromosomes congressed on the metaphase plate (T/C), image (**B**) shows an oocyte with a barrel-shaped spindle and congressed chromosomes (B/C), image (**C**) shows an oocyte with a disintegrated spindle and congressed chromosomes (D/C), and image (**D**) shows an oocyte with a disintegrated spindle and scattered chromosomes (D/S). Graphs (**E**) and (**F**) show percentages of oocytes with different spindle/chromosome configurations following oocytes were aged with different concentrations of sTNF-α or sFasL, respectively. Each treatment was repeated 3-4 times with each replicate containing about 20 oocytes. a,b: Values with a different letter above bars differ significantly (P<0.05) within spindle/chromosome morphologies.

To observe spindle/chromosome morphology, newly ovulated DOs were first aged for 12 h in CZB containing different concentrations of sTNF-α or sFasL, then cultured for 24 h in CZB medium. At the end of the second culture, oocytes were examined for morphology of spindles and chromosomes by confocal microscopy. The results (Fig. 5) showed that while treatment with 5 ng/ml sTNF-α decreased the proportion of normal oocytes with tine pole spindle and congressed chromosomes while increasing that of abnormal oocytes with disintegrated spindle and congressed chromosomes significantly (Fig. 5E), the proportion of normal oocytes did not change significantly until the concentration of sFasL increased to 10 ng/ml (Fig. 5F). Taken together, the results further confirmed that both sTNF-α and sFasL facilitated late manifestations of oocyte aging, and that aging mouse oocytes were more sensitive to sTNF-α than to sFasL in vitro.

### Contents of sTNF-α and sFasL in CM conditioned with CCs for different times

**Figure 6:**
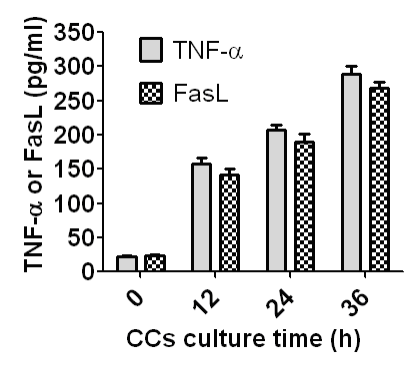
**Concentrations of sTNF-α and sFasL in CM conditioned with CCs for different times.** CCs recovered from newly ovulated oocytes were cultured in CZB medium containing 200-µM H_2_O_2_, and at different times of the culture, CM was recovered for ELISA measurement of sTNF-α and sFasL. One CM sample recovered was divided into two parts: one part for sTNF-α and the other part for sFasL measurement. Each treatment was repeated 3-4 times and each replicate contained 100 µl of CM from one culture well. Difference between sTNF-α and sFasL concentrations was insignificant (P>0.05) at all time points observed.

The CCs collected from newly ovulated oocytes recovered at 13 h post hCG injection were cultured in CZB medium containing 200 µM H_2_O_2_, and at different times of the culture, concentrations of sTNF-α and sFasL in CM were measured by ELISA. No significant difference in concentrations was observed between sTNF-α and sFasL at all the time points examined (Fig. 6), suggesting that the apoptotic CCs released the same amount of sTNF-α and sFasL during oocyte aging. The results further substantiated the conclusion that CCs facilitated oocyte aging by releasing both sTNF-α and sFasL.

### Experiments using TNF-α-knockdown CCs and the COCs from TNF-α-knockout mice

**Figure 7:**
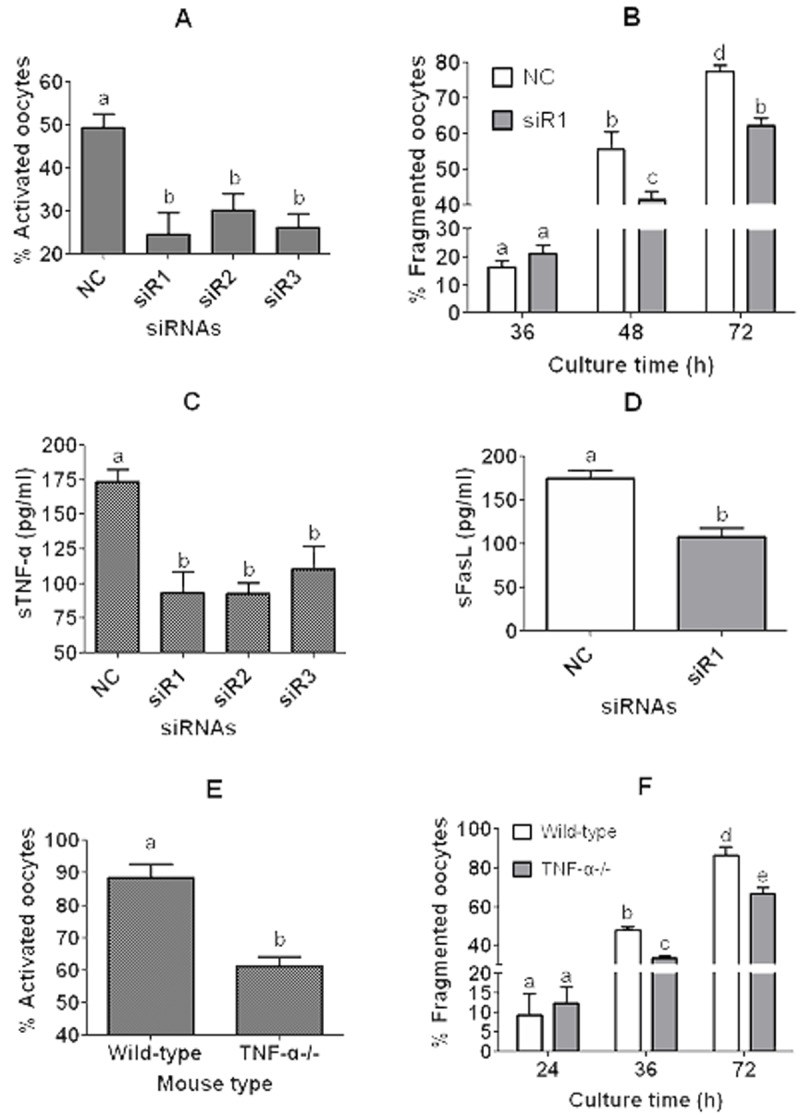
**Experiments using TNF-α-knockdown CCs and the COCs from TNF-α-knockout (TNF-α^-/-^) mice.** In panels (**A**) to (**D**), CCs were transfected with negative control siRNA (NC) or siRNAs (siR) 1, 2 or 3 against TNF-α before culture in CZB containing 200-µM H_2_O_2_ and CM collection. In panels (**A**) and (**B**), DOs recovered 13 h post hCG injection were cultured in the CM for 12 h before ethanol treatment for activation (**A**) or culture in CZB for different times for fragmentation observation (**B**). In panels (**C**) and (**D**), concentrations of sTNF-α or sFasL in the CM were measured by ELISA, respectively. In panels (**E**) and (**F**), COCs recovered 13 h post hCG injection from wild-type C57BL/6J mice and TNF-α^-/-^ mice were cultured for 12 h in CZB medium before examination for ethanol activation (**E**) or for fragmentation (**F**). To observe fragmentation, the COCs were freed of CCs and the resulting DOs were cultured in CZB for different times before examination for fragmentation. To examine activation or fragmentation, each treatment was repeated 3-5 times with each replicate including about 30 oocytes. For measurement of sTNF-α or sFasL in CM, each treatment was repeated 3 times with each replicate including 100 µl of CM from one culture well. a-e: Values with a different letter above bars differ significantly (P < 0.05).

To further verify that CCs facilitate oocyte aging by secreting sTNF-α, two experiments were conducted using TNF-α-knockdown CCs and the COCs from TNF-α-knockout mice, respectively. In the first experiment, expression of TNF-α in CCs was knocked down by RNA interference before CM preparation. Then, DOs recovered 13 h post hCG injection were cultured in the CM for 12 h before examination for rates of ethanol activation or fragmentation. The results showed that oocyte activation rates were significantly lower after culture in CM produced by CCs transfected with any of the three small interfering RNAs against TNF-α than in CM conditioned with CCs transfected with negative control (NC) siRNAs (Fig. 7A). To observe fragmentation rates, the DOs that had been treated for 12 h in CM were cultured in CZB for different times before examination for fragmentation. Up to 36 h of the CZB culture, oocyte fragmentation rates did not differ between the NC siRNA and siRNA-1 groups, but after 48 h of the CZB culture, the fragmentation rate was significantly lower in the siRNA-1 group than in the NC siRNA group (Fig. 7B). Our measurement of sTNF-α in CM indicated that CCs transfected with any of the three small interfering RNAs released significantly less sTNF-α than the CCs transfected with NC siRNAs did (Fig. 7C). Our measurement of sFasL in CM demonstrated that CCs transfected with siRNA-1 produced significantly less sFasL than the CCs transfected with NC siRNA did (Fig. 7D).

In the second experiment, COCs recovered 13 h post hCG injection from wild-type C57BL/6J mice and TNF-α^-/-^ mice were cultured for 12 h in CZB medium before examination for rates of ethanol activation or fragmentation. Activation rates of COCs from TNF-α^-/-^ mice were significantly lower than those of COCs from wild-type C57BL/6J mice (Fig. 7E). To observe fragmentation rates, the COCs that had been cultured for 12 h in CZB were freed of CCs and the resulting DOs were cultured in CZB for different times before fragmentation examination. Up to 24 h of the DO culture, oocyte fragmentation rates did not differ between the wild-type and TNF-α^-/-^ mice, but after 36 h of the DO culture, the fragmentation rate was significantly lower in the TNF-α^-/-^ mice than in the wild-type mice (Fig. 7F). Taken together, the results further confirmed that CCs facilitate oocyte aging by secreting sTNF-α and that TNF-α facilitates sFasL secretion in aging CCs.

## DISCUSSION

The present results showed that the TNF-α signaling was well established in ovulated mouse oocytes. Thus, the CCs underwent apoptosis and secreted sTNF-α, and the oocytes expressed a full complement of TNF-R1 during in vitro aging after ovulation. Expression of TNF-α in CCs and TNF-R in the oocyte has been reported in several mammalian species. For example, Deb et al. [19] observed transcripts of TNF-α and TNF-R1 in bovine CCs together with those of some apoptosis-related genes. Johnson et al. [20] found that TNF-α was confined mainly to the ovine oocyte before GnRH administration, but accumulated in CCs during the mid-to-late preovulatory period. Naz et al. [21] reported that human oocytes and CCs expressed TNF-α and TNF-R1 and 2, both at the mRNA and protein levels. Furthermore, Marcinkiewicz et al. [15] observed that TNF-R was localized on oocytes, granulosa cells and interstitial cells in the rat ovary. Although it is generally accepted that TNF-R is a surface receptor, the present results showed that TNF-R1 was localized in oocyte cytoplasm as well as the plasma membrane. Our previous study also observed Fas localization in both plasma membrane and cytoplasm of mouse oocytes [6]. Furthermore, TNFR1 localization in cytoplasm and in the nucleus has been observed in large diameter and small diameter dorsal root ganglion neurons, respectively [22].

In this study, we observed that the concentration of TNF-α in CM conditioned with CCs increased with conditioning time. Oocytes cultured in the CM showed a significant increase in activation rate but a significant decrease in MPF activity with increasing conditioning time. After culture of oocytes in the presence of optimal concentrations of sTNF-α, while blastocyst rates decreased, rates of activation, fragmentation and abnormal spindles increased significantly. Furthermore, the aging-promoting effect of sTNF-α was significantly reduced in TNF-α knockdown CCs and in CCs from the TNF-α-knockout mice. Taken together, the results strongly suggested that CCs promoted oocyte aging in a paracrine manner by releasing sTNF-α. One of the important mechanisms operative in apoptosis is the so-called cell surface receptor-mediated apoptosis triggered by the binding of death molecule (TNF and Fas) ligand to cell surface receptors [23]. The possibility of autocrine or paracrine actions of TNF as an intra-ovarian regulator of oocyte apoptosis has been suggested in the neonatal rat [15]. When bovine oocytes were matured with various concentrations of TNF-α, the percent of oocytes that developed to the blastocyst stage after insemination was reduced significantly at all the TNF-α concentrations tested [16]. Furthermore, exposure of porcine oocytes to elevated concentrations of TNF-α significantly reduced their maturation rates while increasing the proportion of oocytes with abnormal chromosome alignment and cytoskeleton structure [24].

In this study, we measured the concentrations of sTNF-α and sFasL in CM conditioned with CCs for different times and found that the apoptotic CCs released equal amounts of sTNF-α and sFasL during oocyte aging. Previous studies demonstrated that the Fas signaling and the TNF-α signaling could be present simultaneously in a system. For example, Cherng et al. [25] observed that treatment of rats with streptozotocin to induce diabetes significantly increased the protein levels of both TNF-α and Fas. Baka and Malamitsi-Puchner [26] reported that both sFas-sFas ligand system and TNF-α were present in the follicular fluid and may influence oocyte developmental potential. Furthermore, Driancourt and Thuel [27] documented that compounds involved in the initiation of apoptosis such as TNF-α and Fas are present in oocytes together with proteins involved in cell death (BAX) or cell survival (BCL2). Previous studies have also indicated that there is crosstalk between the Fas and TNF systems and that TNF-α can promote expression of FasL and Fas, and vice versa. For example, TNF-α increased expression of both FasL and Fas in astrocytes [28], and promoted Fas expression in Sertoli cells [29]. Furthermore, FasL can facilitate expression of TNF-α in macrophages [30]. However, there are papers reporting that TNF-alpha inhibited expression of Fas in the prostate cancer cells [31]. Thus, whether the conflicting results reported by different papers on the effect of TNF-α on FasL/Fas expression are due to different cell types or other factors is in question. Our present observations confirmed that TNF-α facilitates sFasL secretion in the aging CCs.

In summary, although previous studies indicated that CCs accelerate oocyte aging by releasing soluble factors, the factors have yet to be characterized. While demonstrating that CCs promoted oocyte aging by releasing sFasL, our recent study suggested that CCs might secrete other factors to mediate oocyte aging as well. Like the Fas system, the TNF-α system is also well known for its ability to induce apoptosis in various cells and tissues. We thus hypothesized that CCs might accelerate oocyte aging by secreting TNF-α. This study has confirmed the hypothesis by showing that aging mouse CCs secreted sTNF-α which accelerated oocyte aging by interacting with TNF-R on the oocyte. Furthermore, the aging CCs produced equal amounts of sTNF-α and sFasL, and TNF-α promoted sFasL production in these cells. The data are important for animal breeding facilities and may be relevant for the human as well, as it has been reported that human oocytes and CCs expressed both TNF-α and TNF-R1/2 [21,32].

## MATERIALS AND METHODS

Animal care and handling were conducted strictly according to the guidelines issued by the Animal Care and Use Committee of the Shandong Agricultural University, P. R. China (Permit number: SDAUA-2001-001). All the chemicals and reagents used were purchased from Sigma Chemical Company unless otherwise mentioned.

### Oocyte recovery

Mice of the Kunming breed, which were bred in this laboratory, were used in most of the experiments in this study. The TNF-α^-/-^ mice with a C57BL/6J genomic background were obtained from Model Animal Research Center of Nanjing University, Nanjing, China. The wild-type C57BL/6J mice were purchased from Shandong University Center for Laboratory Animals. The mice were kept in a room under a 14L:10D photoperiod, with lights-off at 20:00. Female mice (6–8 weeks of age) were superovulated with 10 IU equine chorionic gonadotropin (eCG, i.p.) and 10 IU human chorionic gonadotropin (hCG, i.p.) at a 48-h interval. Both eCG and hCG were purchased from Ningbo Hormone Product Co., Ltd., China. The superovulated mice were sacrificed 13 h after hCG injection, and COCs were recovered from the oviducts. After dispersing and washing three times in M2 medium, the COCs were denuded of CCs by pipetting with a thin pipette in a drop of M2 medium containing 0.1% hyaluronidase to prepare DOs.

### Preparation of CCs and conditioned medium (CM)

The CCs isolated during the preparation of DOs were washed twice by centrifugation (200× g, 5 min each) in regular CZB medium. The pellets were then resuspended in a proper volume of CZB containing 200 µM H_2_O_2_ to obtain a final suspension of 5–8×10^5^ CCs/ml. The H_2_O_2_ was used because our previous study had shown that the presence of H_2_O_2_ at this concentration significantly increased the apoptotic rates and sFasL secretion of the CCs [6]. The cell suspension was then added to wells of a 96-well culture plate (200 µl per well) and incubated at 37.5°C for 24 h in a humidified atmosphere containing 5% CO_2_. At the end of the treatment, CCs were harvested and washed by centrifugation. The resultant pellets were resuspended in regular CZB, and the cell suspension was incubated at 37.5°C for 24 or 36 h in a humidified atmosphere containing 5% CO_2_. At the end of the incubation, CM was recovered and centrifuged at 300×g for 5 min to remove cells and debris. The CM obtained was frozen at −80°C until use.

To prepare CM from TNF-α-knockdown CCs, CCs were transfected with siRNAs. The siRNAs targeting mRNAs and the negative control siRNA were designed and synthesized by RiboBio (Guangzhou, China). The sense strands of targeting siRNAs for TNF-α gene included siRNA-1 (5'-GAC AAC CAA CTA GTG GTG C-3'), siRNA-2 (5'-CCA ACG GCA TGG ATC TCA A-3', siRNA-3 (5'-CGT CGT AGC AAA CCA CCA A-3'), and siR-RiboTM for negative control. Transfection with 100 nM siRNAs was conducted using lipofectamine RNAiMAX reagent (Invitrogen/Life Technologies, Grand Island, NY). Briefly, 0.5 µl of a 20 µM solution of each siRNA were diluted in 4.5 µl of Opti-MEM medium (Invitrogen) and mixed with 0.3 µl of Lipofectamine RNAiMAX reagent (Invitrogen) in 4.7 µl of Opti-MEM medium. After incubation for 5 min at room temperature, the transfection complex was mixed with 90 µl regular CZB medium to obtain transfection culture medium. Then, freshly recovered CCs were resuspended in proper volume of the transfection culture medium to obtain a final suspension of 5–8×10^5^ CCs/ml. The cell suspension was incubated at 37.5°C for 48 h in a humidified atmosphere containing 5% CO_2_. At the end of the transfection, the CCs were cultured for 24 h in CZB containing 200-µM H_2_O_2_ and then incubated in regular CZB for 24 h before CM collection.

### In vitro aging of oocytes

For in vitro aging, DOs were cultured in CM or regular CZB medium supplemented with different concentrations of sTNF-α or sFasl. Briefly, CM or CZB with sTNF-α or sFasl was placed in wells of a 96-well culture plate (100 ml per well). About 30 DOs were transferred to each well, covered with mineral oil, and cultured for different times at 37.5°C under 5% CO_2_ in humidified air. Stock solutions of recombinant mouse sFasL (100 µg/ml) and recombinant mouse TNF-α (100 µg/ml) were prepared by dissolving the recombinant sFasL (R&D System) or TNF-α (R&D System) in PBS containing 0.1% bovine serum albumin. The stock solutions were stored at −20°C and were diluted to desired concentrations with CZB immediately before use.

### Assessment of CCs apoptosis

The CCs obtained from 60-90 freshly recovered COCs or from one well of cell culture were separated by centrifugation (200×g, 5 min at room temperature). For Hoechst staining, the pellets of CCs were resuspended in 50 µl of M2 medium supplemented with 10 µg/ml of Hoechst 33342 and stained in the dark for 5 min. For annexin and Hoechst double staining, an Annexin V-FITC apoptosis detection kit (KGA105-KGA108, KeyGEN BioTECH) was used. Briefly, 5 µl of Annexin V-FITC were mixed with 500 µl Binding buffer. Then, the CCs pellets were resuspended in the above mixture and stained in the dark for 20 min. After centrifugation for 5 min at 200×g, the CCs pellets were resuspended in 50 µl of M2 medium supplemented with 10 µg/ml of Hoechst 33342 and stained in the dark for 5 min. The stained cells were then centrifuged for 5 min at 200×g. After about half of the supernatant was removed, a 5-µl drop of suspension was smeared on a slide and the smear was observed under a Leica DMLB fluorescence microscope at a 400× magnification. On the Hoechst-stained smears, the heterochromatin was heavily stained with Hoechst giving bright fluorescence. Whereas the apoptotic CCs showed pyknotic nuclei full of heterochromatin, healthy CCs displayed normal nuclei with sparse heterochromatin spots. On the annexin and Hoechst double-stained smears, cells with more than 50% of the plasma membrane showing green fluorescence were considered annexin-positive. On each smear, 6-8 fields were randomly examined and the percentages of apoptotic cells were calculated from 60–80 CCs in each field. All images were reviewed by 2 investigators in a double blind manner.

### Enzyme-linked immunosorbent assay (ELISA) for sTNF-α and sFasL in CM

Contents of sTNF-α and sFasL in CM were measured by ELISA using a mouse tumor necrosis factor α (TNFα) Elisa kit (BLUE GENE, Shanghai, China) and a mouse factor related apoptosis ligand (FASL) Elisa kit (BLUE GENE, Shanghai, China), respectively. Briefly, 100 µl of standards or samples were added in duplicate to wells of a micro-titer plate pre-coated with mouse monoclonal antibodies, then 50 µl of conjugate was added to each well and incubated for 60 min at 37°C. After the micro-titer plate was washed using the wash solution and dried using paper towels, 50 µl of substrate A and 50 µl of substrate B were added to each well and incubated for 15 min at 25°C. The optical density was measured at 450 nm using a plate reader (BioTek-ELx808, BioTek Instruments, Inc.) within 15 min after the reaction was terminated by 50 µl of stop solution. The concentrations of sTNF-α and sFasL in CM were calculated against the respective standard curves.

### Immunofluorescence microscopy for detection of TNF-R1 and tubulin

All the detection procedures were conducted at room temperatures unless otherwise mentioned. DOs were washed three times in M2 between procedures. DOs were (1) fixed for 30 min in PBS with 3.7% paraformaldehyde; (2) zona pellucida were removed by a 10-second treatment with 0.5% protease in M2; (3) permeabilized with 0.1% Triton X-100 in PBS at 37.5°C for 10 min; and (4) blocked with 3% BSA in PBS at 37.5°C for 30 min. For TNF-R1 detection, the blocked oocytes were first incubated at 4°C overnight with rabbit polyclonal anti-TNF-R1 (IgG, 1:100, Abcam) in 3% BSA in M2 medium, and then, with Cy3-conjugated goat-anti-rabbit IgG (1:800, Jackson ImmunoResearch) in 3% BSA in M2 for 1 h. Finally, the oocytes were incubated for 10 min with 10 µg/ml Hoechst 33342 in M2 to stain chromatin. Samples in which the primary antibody was omitted were also processed to serve as negative controls. For tubulin staining, the blocked DOs were first incubated at 37°C for 1 h in PBS containing FITC-conjugated anti-α-tubulin monoclonal antibodies (1:50), and then, incubated for 10 min with 10 µg/ml Hoechst 33342 in M2 to stain chromatin. The stained oocytes were mounted on glass slides as follows. Oocytes were transferred with as little accompanying medium as possible to the center of 4 wax spots on a microscopic slide. The wax was made of 15 parts of vaseline, 1 part of paraffin and a small amount of bee wax; mixed by melting and applied to the slide with a needle. The oocytes were then pushed together with a needle, compressed moderately under a coverslip, and observed with a Leica laser scanning confocal microscope (TCS SP2). Blue diode (405 nm), argon (488 nm), and helium/neon (543 nm) lasers were used to excite Hoechst, FITC, and Cy3, respectively. Fluorescence was detected with bandpass emission filters: 420–480 nm for Hoechst, 505–540 nm for FITC, and 560–605 nm for Cy3, and the captured signals were recorded as blue, green and red, respectively.

### Western blot analysis

About 200 DOs were placed in a 1.5 ml microfuge tube containing 20 µl sample buffer (20 mM Hepes, 100 mM KCl, 5 mM MgCl_2_, 2 mM DTT, 0.3 mM PMSF, 3 mg/ml leupetin, pH 7.5) and frozen at −80°C until use. For running the gel, 5 µl of 5× SDS-PAGE loading buffer was added to each tube and the tubes were heated at 100°C for 5 min. The samples were separated on a 12% SDSPAGE and transferred onto PVDF membranes. The membranes were washed twice in TBST (150 mM NaCl, 2 mM KCl, 25 mM Tris, 0.05% Tween-20, pH 7.4) and blocked for 1.5 h with TBST containing 3% BSA at room temperature. The membranes were then incubated at 4°C overnight with primary antibodies at a dilution of 1:1000 in 3% BSA-TBST. After being washed three times in TBST (5 min each), the membranes were incubated for 1 h at 37°C with second antibodies diluted to 1:2000 in 3% BSA-TBST. After washing 3 times in TBST, the membranes were detected by a BCIP/NBT alkaline phosphatase color development kit (Beyotime Institute of Biotechnology, China). The relative quantities of proteins were determined by analyzing the sum density of each protein band image using Image J software. GAPDH was used as internal controls. The primary antibodies used included rabbit anti-TNF-R1 polyclonal antibody (Abcam, ab19139) and mouse anti-GAPDH monoclonal antibody (ComWin Biotech, CW0100M). The secondary antibodies included goat anti-rabbit IgG AP conjugated (ComWin Biotech, CW0111) and goat anti-mouse IgG AP conjugated (ComWin Biotech, CW0110).

### Oocyte activation

Two activation protocols were used in this study: ethanol plus 6-dimethylaminopurine (6-DMAP) was used to assess oocyte activation susceptibility, while SrCl_2_ was used to evaluate oocyte developmental potential. During the ethanol+6-DMAP treatment, oocytes were first treated with 5% ethanol in M2 medium for 5 min at room temperature, and then, cultured in CZB medium containing 2 mM 6-DMAP for 6 h at 37.5°C in a humidified atmosphere containing 5% CO_2_ in air. For SrCl_2_ activation, oocytes were incubated for 6 h in Ca^2+^-free CZB medium supplemented with 10 mM SrCl_2_ and 5 µg/ml cytochalasin B. At the end of the activation culture, oocytes were observed under a inverted microscope for activation. Oocytes showing one or two pronuclei, or showing two cells each having a nucleolus, were judged as activated.

### Embryo culture

The Sr^2+^-activated oocytes were cultured in regular CZB medium (about 30 oocytes per well containing 100 µl medium) at 37.5°C under a humidified atmosphere with 5% CO_2_ in air. On day 2 of culture, oocytes were examined for 4-cell development, and were transferred to CZB medium containing 5.55 mM glucose for further culture. On day 4.5 of the culture, oocytes were examined for blastocyst formation.

### Assessment of oocyte fragmentation

At different times of the aging culture, DOs were examined under a phase contrast microscope for morphological feature. Oocytes with a clear moderately granulate cytoplasm, and an intact first polar body, were considered un-fragmented. Oocytes with more than two asymmetric cells were considered fragmented.

### Maturation-promoting factor (MPF, p34^cdc2^ kinase) assay

The MPF (p34^cdc2^ kinase) activity was assayed using a MESACUP cdc2 kinase assay kit (code 5234; MBL, Nagoya, Japan). Briefly, about 50 DOs were placed in a plastic tube containing 10 µl of cell lysis buffer (50 mM Tris [pH 7.5], 150 mM NaCl, 2 mM EDTA, 5 mM EGTA, 1% [v/v] Triton X-100, 2.5 mM sodium pyrophosphate, 1 mM β-Glycerophosphate, 1 mM Na_3_VO_4_, 1 µg/ml leupeptin, and 1 mM PMSF). The tubes were then frozen at −80°C and thawed three times at room temperature. The resultant cell extracts were stored at −80°C until use. To conduct the assay, 10 µl of oocyte extracts were mixed with 35 µl of kinase assay buffer B (25 mM Hepes buffer [pH 7.5, MBL], 10 mM MgCl_2_ [MBL], 10% [v/v] MV peptide solution [SLYSSPGGAYC, MBL], 0.1 mM ATP), and the mixture was incubated for 30 min at 30°C. Then 200 ml PBS containing 50 mM EGTA (MBL) were added to terminate the reaction. The phosphorylation of MV peptides was detected at 492 nm using a plate reader (BioTek-ELx808, BioTek Instruments, Inc.). The value of MPF activity in the newly ovulated control oocytes was set as 100% and the other values were expressed relative to this value.

### Data analysis

In all the experiments, each treatment was repeated at least three times. Data were arcsine transformed before being analyzed with ANOVA. A Duncan multiple test was performed to determine the differences. The software of SPSS 11.5 (SPSS Inc.) was used. Data are expressed as mean ± SEM, and P < 0.05 was considered significant.
